# Effects of Alloying Elements on the Formation of Core-Shell-Structured Reinforcing Particles during Heating of Al–Ti Powder Compacts

**DOI:** 10.3390/ma11010138

**Published:** 2018-01-15

**Authors:** Tijun Chen, Min Gao, Yunqi Tong

**Affiliations:** State Key Laboratory of Advanced Processing and Recycling of Nonferrous Metals, Lanzhou University of Technology, Lanzhou 730050, China; gaom199409@163.com (M.G.); tongyq1991@163.com (Y.T.)

**Keywords:** powder thixoforming, Al matrix composite, core–shell structured reinforcing particles, phase constituent, microstructure

## Abstract

To prepare core-shell-structured Ti@compound particle (Ti@compound_p_) reinforced Al matrix composite via powder thixoforming, the effects of alloying elements, such as Si, Cu, Mg, and Zn, on the reaction between Ti powders and Al melt, and the microstructure of the resulting reinforcements were investigated during heating of powder compacts at 993 K (720 °C). Simultaneously, the situations of the reinforcing particles in the corresponding semisolid compacts were also studied. Both thermodynamic analysis and experiment results all indicate that Si participated in the reaction and promoted the formation of Al–Ti–Si ternary compounds, while Cu, Mg, and Zn did not take part in the reaction and facilitated Al_3_Ti phase to form to different degrees. The first-formed Al–Ti–Si ternary compound was τ1 phase, and then it gradually transformed into (Al,Si)_3_Ti phase. The proportion and existing time of τ1 phase all increased as the Si content increased. In contrast, Mg had the largest, Cu had the least, and Si and Zn had an equivalent middle effect on accelerating the reaction. The thicker the reaction shell was, the larger the stress generated in the shell was, and thus the looser the shell microstructure was. The stress generated in (Al,Si)_3_Ti phase was larger than that in τ1 phase, but smaller than that in Al_3_Ti phase. So, the shells in the Al–Ti–Si system were more compact than those in the other systems, and Si element was beneficial to obtain thick and compact compound shells. Most of the above results were consistent to those in the semisolid state ones except the product phase constituents in the Al–Ti–Mg system and the reaction rate in the Al–Ti–Zn system. More importantly, the desirable core-shell structured Ti@compound_p_ was only achieved in the semisolid Al–Ti–Si system.

## 1. Introduction

It is known that intermetallic particles, such as Al_3_Fe, Al_3_Ti, and Al_3_Ni that can in situ form through reaction during solidification of Al melts, have a good wettability and close coefficient of thermal expansion with Al matrix alloys, and thus, clean and strong interface can be obtained and they are considered as ideal reinforcements for Al matrix composites [1,2,3,4]. Among them, Al_3_Ti is more attractive because of its superior properties, such as low density, high melting point and Young’s modulus, and good thermodynamical stability in Al alloys [5]. Chen et al. have fabricated Al_3_Ti particle (Al_3_Ti_p_) reinforced Al matrix composite by direct reaction of Ti–containing fluxes with Al melt [6]. The needed high temperature (1273 K (1000 °C)) resulted in serous gas absorption and oxidation. Chianeh et al. have producedAl_3_Ti_p_/Al matrix composite by powder metallurgy (PM) [7]. The resulting composite inevitably contained numerous pores and still needed subsequent plastic processing although the distribution of Al_3_Ti_p_ was quite uniform and the constituents could be flexibly designed. In addition, this technology is relatively difficult to fabricate components with large size and complicated shape. Nevertheless, as a promising processing technology of alloys, thixoforming, not only can significantly decrease or eliminate pores, but is also appropriate for producing large components with complex shapes [8]. By combining the merits of both PM and thixoforming, a novel technology that integrates the preparation and forming Al_3_Ti_p_/Al matrix composites, named powder thixoforming, has been proposed by the authors [9,10]. A green compact is first obtained by blending and cold pressing of Ti and Al alloy powder mixture. Then the compact is partially remelted and thixoformed. Through partial remelting, not only the semisolid nondendritic ingot that is needed by thixoforming can be achieved, but also Al_3_Ti reinforcing particles can be in situ generated through reaction between the Ti powders and Al melt.

However, Al_3_Ti_p_, similar to the other ceramic or intermetallic particles, can significantly improve tensile strength, but sharply decrease ductility of the composites [7,11]. It can be expected that if the reinforcing particles have a core-shell structure with a soft Ti core and a hard Al_3_Ti intermetallic shell (expressing this kind of reinforcements as Ti@Al_3_Ti_p_), they should have a good strengthening role that is similar to that of monolithic Al_3_Ti particles when the thickness of the Al_3_Ti shell reaches a critical value, but cracks that first generate in the Al_3_Ti phase during tensile test are constrained within the shell, and thus, the crack sizes must be obvious smaller than those generated in the monolithic Al_3_Ti_p_ with a same size to the core-shell-structured particles. In addition, two tips of each crack face the soft Ti core and Al matrix, respectively, and subsequent propagation will be tentatively prevented or delayed by blunting of the crack tips. The propagation does not operate until a given serious plastic deformation has occurred in both the Ti core and Al matrix. So, as expected, the ductility of the composite is improved. Song et al. prepared a core-shell-structured Fe@Al_x_Fe_y_ particle reinforced Al matrix composite by PM [12]. The results indicated that the compressive strength of the composite was obviously enhanced in comparison with the Al matrix alloy and the compressive ductility was also acceptable [12]. But, the tensile properties were very poor and the tensile strain was less than 1% due to lots of pores and voids resulted from the solid reaction and processing characteristics of this technology. Subsequently, they fabricated Ti@Al_3_Ti_p_/Al matrix composite using the same technology, the tensile properties of the resulting composite were improved, but numerous small pores were still existed [13]. It can be expected that the pores can be filled to some extent by liquid phase under high pressure if powder thixoforming is applied, and thus, the tensile properties, especially the ductility, can be further improved. Our preliminary investigation indicated that the Ti@(Al–Ti–Si)_p_/A356 matrix composite prepared by powder thixoforming had an excellent elongation of 8.3%, increased by 168% when compared to the (Al,Si)_3_Ti_p_/A356 composite, which was thixoformed after the Ti powders had completely reacted [14]. In addition, it had comparable tensile yield and ultimate strengths to the latter composite. This indicates that the powder-thixoforming is a promising way to fabricate the Ti@Al_3_Ti_p_/Al matrix composite with higher performance, especially high ductility.

The previous investigation also indicated that the microstructure of the Ti@Al_3_Ti_p_, such as the thickness and compactness of the Al_3_Ti shells, had large effect on the strengthening and toughening roles to the Al matrix alloy [14]. Compact shells with a given thickness should be the prerequisite for achieving excellent mechanical properties. Our earlier investigations disclosed that the reaction intermetallic shells formed during partial remelting of A356–Ti green compact at 868 K (595 °C)were thicker and compacter than those of 2024-Ti compact at 913 K (640 °C) [9,10]. In addition, the phase constituent of the reaction shells in the A356-Ti system varied as the partial remelting proceeded due to the participation of Si element in the reaction [9,14], while that in the 2024-Ti compact always maintained a unique phase of Al_3_Ti and Cu element did not take part in the reaction [10]. That is, the alloying elements have obvious effects on the formation and microstructure of the core-shell-structured reinforcing particles. Unfortunately, this problem can only be found through comparing these two reports, and the detailed influences, including the effects of the other alloying elements have not been reported till now. Therefore, the effects of the commonly-used alloying elements in Al alloys, such as Si, Cu, Mg, and Zn, on the reaction between the Ti powders and Al melt, and the formation and microstructure of the resulting core-shell-structured particles were investigated from thermodynamic calculations and experiment results, in order to provide theoretical and experimental foundations for achieving ideal Ti@compound core-shell-structured reinforcing particles during powder thixoforming of Al matrix composites.

## 2. Thermodynamic Calculations

### 2.1. Miedema’s Model and Gibbs Free Energy

To predict the reaction products, Miedema’s model, a semi–empirical model to estimate the affinity between alloying elements, was used in this work. The possibility for forming a compound between two elements can be predicted according to their Miedema formation enthalpy values. The more negative the formation enthalpy is, the greater the affinity is, and thus, the larger the possibility for forming a compound. In the past decades, this theory has been widely applied to estimate the formation enthalpy of liquid and solid phases in over 500 binary alloys. The deviation between the calculated and experimental values generally does not exceed 8 kJ/mol [15]. According to the first principles, this model can be used for any alloy; however, it is usually very complicated and requires lots of approximations and assumptions for ternary and higher alloys [16,17]. Therefore, Miedema’s model is always used in binary alloys.

In the Miedema’s model, the formation enthalpy, Δ*H_ij_* in a binary *i-j* alloy system can be expressed as the following Equation [18]:

(1) ΔHij=fijxi[1+μixj(φi−φj)]xj[1+μjxi(φj−φi)]xiVi23[1+μixj(φi−φj)]+xjVj23[1+μjxi(φj−φi)]

and *f_ij_* is defined as

(2) fij=2pVi23Vj23{qp[(nWS13)j−(nWS13)i]2}−(φj−φi)2−α(rp)(nWS13)i−1+(nWS13)j−1

where *V*, *φ* and *n_ws_* are the molar volume, chemical potential, and electron density of an element, respectively, *x* is the mole fraction of each constituent and *p*, *q*, *r* and α are empirical constants. For all alloys, *q*/*p* equals to 9.4V^2^/(density unit)^2/3^. The *p* equals to 12.3 for an alloy composed of a transition element and a non-transition element, to 14.1 for an alloy consisted of two transition elements, and to 10.6 for an alloy having two non–transition elements. For an alloy composed of a transition element and a non-transition element, the *r/p* equals to the product of the two elements’ *r/p* values, and equals to 0 for other alloys. The α equals to 0.73 for liquid alloys. The values of the other parameters can be found in Table 1.Using Equations (1) and (2), the formation enthalpy values for the binary alloys Al–X (X = Si, Cu, Mg, Zn, or Ti) and X–Ti (X = Si, Cu, Mg, or Zn) were calculated.

**Figure 1:**
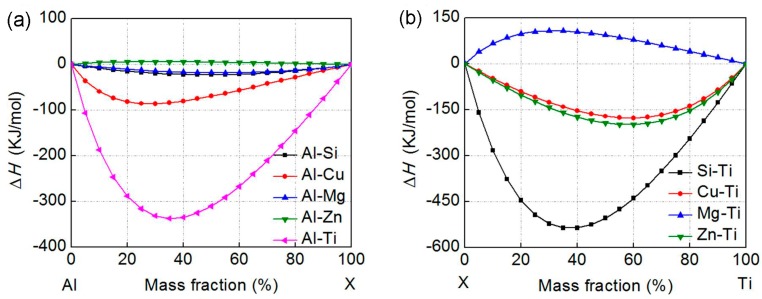
Variations of formation enthalpy with alloying element contents. (**a**) Al–X (X = Si, Cu, Mg, Zn, and Ti) systems and (**b**) X–Ti (X = Si, Cu, Mg, and Zn) systems.

The calculation results are shown in Figure 1. It can be found that the Al–Ti system has the lowest formation enthalpy among the five systems and the formation enthalpy for the other four systems increases in a sequence of Al–Cu, Al–Si, Al–Mg, and Al–Zn (Figure 1a). As indicated by Figure 1b, the Si–Ti system has the lowest formation enthalpy among the four X–Ti (X = Si, Cu, Mg, or Zn) systems and the formation enthalpy for the other three systems increases in turn of Zn–Ti, Cu–Ti, and Mg–Ti. In addition, the formation enthalpy of Si–Ti system is lower than that of Al–Ti system (comparing Figure 1a,b).

For Al–Ti–X (X = Si, Cu, Mg, and Zn) ternary systems, the possible binary compounds involve Al–X, X–Ti, and Al–Ti. In view of the above calculations, Si–Ti compound should first precipitate, and then Al–Ti, Zn–Ti, Cu–Ti, Al–Cu, Al–Si, and Al–Mg compounds may in turn form during solidification. But, Al–Zn and Mg–Ti compounds cannot generate owing to their positive formation enthalpy values (Figure 1). Specially, the affinities of Ti with Si and Al are all quite large, although the affinity of Ti with Si is higher than that of Ti with Al. This means that Al–Ti–Si ternary compounds can possibly form in the Al–Si–Ti system. A previous investigation indicated that Al element had large effect on the products of high-temperature self-propagation synthesized Al–Ti–Si alloys [19].The final compound was Ti_5_Si_3_phase when Al content was less than 15 wt %,but was (Al,Si)_3_Ti phase as the Al content exceeded 30 wt %. This further verifies the possibility of forming Al–Ti–Si ternary compounds in the Al–Si–Ti ternary Al–based alloys, i.e., Si element may participate in the reaction between Ti and Al. In addition, Zn–Ti and Cu–Ti binary compounds, or Al–Ti–Zn and Al–Ti–Cu ternary compounds are rarely found in Al alloys although the affinities of Ti with Zn and Cu are all quite large. This implies that these binary or ternary compounds possibly cannot form in spite of the thermodynamic feasibility. Therefore, it can be proposed that the reaction products with Ti should be only Al–Ti binary compounds in the Al–Ti–X (X = Cu, Mg and Zn) systems.

In general, five compounds, such as AlTi_3_, AlTi, Al_2_Ti, Al_2_Ti_5_, and Al_3_Ti, can form in Al–Ti binary alloys [20]. Table 2 gives the Gibbs free energies for forming the five Al–Ti compounds at 993 K [21], which shows that the Gibbs energy increases in a sequence of Al_2_Ti, Al_5_Ti_2_, Al_3_Ti, AlTi_3_, and AlTi. That is, Al_2_Ti and Al_5_Ti_2_ can more easily form than Al_3_Ti. However, as a precursor for forming Al_2_Ti and Al_5_Ti_2_, AlTi cannot precipitate more preferentially than Al_3_Ti at this temperature [22]. So, Al_3_Ti is the unique compound in the Al–Ti binary system at 993 K (720 °C). That is, the reaction products with Ti in Al–Ti–X (X = Cu, Mg and Zn) ternary systems only involve Al_3_Ti phase.

On the basis of the above analysis, it can be proposed that Al_3_Ti is the unique Ti–containing product for the Al–Ti–X (X = Cu, Mg or Zn) ternary systems, while Al–Ti–Si ternary compounds may form in the Al–Ti–Si system due to the high affinities of Ti with Al and Si. That is, Si element may participate in the reaction between Ti and Al, but Cu, Mg, and Zn elements do not involve.

### 2.2. JMatPro Calculations

**Figure 2:**
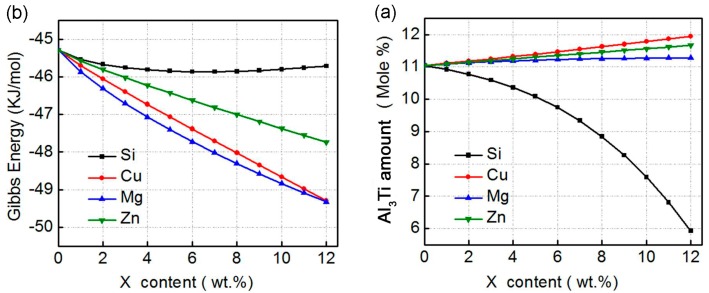
Variations of (**a**) Al_3_Ti amount and (**b**) Gibbs energy with alloying element content in Al–Ti–X (X = Si, Cu, Mg, and Zn) systems at 993K (720 °C).

Moreover, JMatPro (version 7.0; Thermotech Company, Guildford, Surrey, UK), a new multi-platform thermodynamic analysis software to predict phase transformations during solidification of an alloy [23,24], was introduced to make further thermodynamic analysis for the effects of the alloying elements on the compound formation in the Al–Ti–X (X = Si, Cu, Mg, and Zn) ternary systems. The *Thermodynamic Properties* module for *Aluminum alloy* was selected for calculating the variations of Al_3_Ti amount and Gibbs free energy with the alloying element contents. The results are presented in Figure 2. It shows that the Al_3_Ti contents slightly increase as the contents of Cu, Mg, and Zn increase, while that significantly decreases with Si content (Figure 2a). The Gibbs energies continuously decrease with increasing Cu, Mg or Zn contents, but then increases when Si content exceeds 7wt % (Figure 2b). These imply that the stability of Al_3_Ti phase in the Al–Ti–X (X = Cu, Mg, or Zn) systems is improved as the element contents increase, while that in the Al–Ti–Si system becomes unstable when Si content exceeds 7wt % (Figure 2b).

When considering the obvious decrease in Al_3_Ti amount with Si content, it is supposed that Al_3_Ti possibly transforms into the other compounds as Si content exceeds a critical value. This is consistent with the result that Al atoms in Al_3_Ti phase can be partially replaced by Si and other phases, such as (Al,Si)_3_Ti and τ1 (Al_5_Ti_7_Si_12_) phases, then generate [25], and also matches the standpoint achieved from the above analysis of Miedema’s model, Al–Ti–Si ternary compounds may form in the Al–Ti–Si system. Fuji et al. investigated the influence of Si element on the mechanical properties of a friction-welded Ti/Al joint and suggested that the segregation of Si element retarded the formation of Al_3_Ti by acting as a barrier to Ti and Al diffusion at the joint interface [26]. So, it can be summarized that Si can prevent the formation of binary Al_3_Ti, but accelerate the generation of Al–Ti–Si ternary compounds. This should be attributed the larger affinity of Ti with Si than with Al, as discussed in Section 2.1.

Based on the above thermodynamics discussion, it can be proposed that Si element may participate in the reaction between Ti powders and Al melt and promotes the formation of Al–Ti–Si ternary compounds, while the other elements (Cu, Mg, and Zn) do not take part in the reaction and facilitate the formation of Al_3_Ti phase to different degrees as their contents increase. The unique Ti–containing compound in Al–Ti–X (X = Cu, Mg and Zn) systems is Al_3_Ti phase. To confirm these proposals, and verify the detailed effects of the alloying elements on the reaction products with Ti, the formation and microstructure of the reinforcing particles and the corresponding influence mechanisms, experiments were carried out in the following sections.

## 3. Experimental Procedure

**Figure 3:**
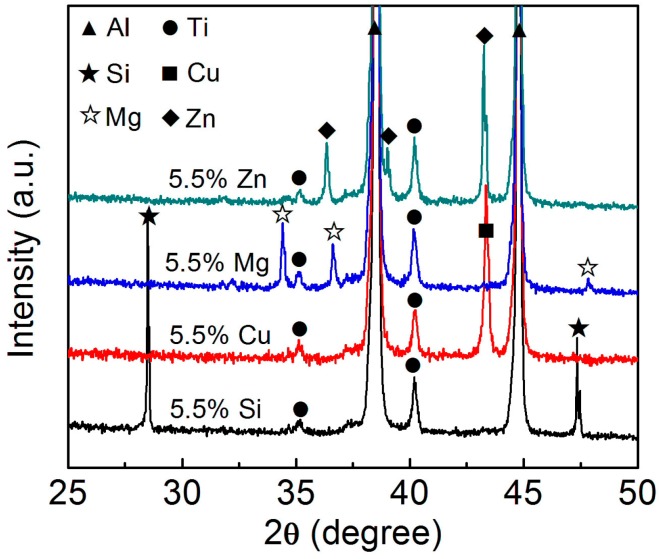
X-ray diffraction (XRD) patterns of the ball-milled Al–5Ti–5.5X (X = Si, Cu, Mg, and Zn) powder mixtures.

The raw materials used in this work include pure Al, Ti, Si, Cu, Mg, and Zn powders, and their purities, average sizes, and preparation methods are given in Table 3. Firstly, certain amounts of Al, Ti powders and one alloying element (Si, Cu, Mg, and Zn) powder were ball-milled for 40 min in a ND7–21 planetary ball-mill machine (Nanjing Levinstep Technology Co., Ltd., Nanjing, China). The employed ball-to-powder ratio and rotation speed were 5:1 and 100 rpm, respectively. Table 4 shows the detailed content of each powder. Ti powder content was kept at 5 wt %, being equivalent to form 10 vol % Al_3_Ti_p_ in the composites. To investigate the effect of one alloying element, the contents of the other three powders were maintained zero. Figure 3 shows the XRD (examined by aD8 ADVANCE X-ray diffraction (XRD; Rigaku, Tokyo, Japan)) patterns of the ball-milled Al–5Ti–5.5X (X = Si, Cu, Mg and Zn) powder mixtures. It indicates that no reactions occurred during ball-milling and the powders in the mixtures still maintained the original pure powders. The obtained powder mixtures were then cold-pressed into green compacts with dimensions of Φ 22 mm × 5 mm by using a XH-300KN pressure machine (Tianjin Xingheng Instrument Factory, Tianjin, China). The employed pressure and holding time were 190 MPa and 5 min, respectively.

The green compacts were heated at 993 K (720 °C) in a tubular vacuum resistance furnace (Tianjin Zhonghuan Furnace Co., Ltd., Tianjin, China) for different durations ranging from 10 min to 30 min, and then quickly water-quenched. Subsequently, each heated compact was cut into two parts along radial direction, and the cross-section of one part was ground, finished, polished, and etched using 10 vol % NaOH aqueous solution. They were then observed and analyzed by a QUANTA FEG 450scanning electron microscope (SEM; FEI, Hillsboro, OR, USA) and an energy dispersive spectroscope (EDS), equipped in the SEM. The phase constituents of the reaction products were examined by the D8 ADVANCEXRD. The thickness of the reaction compound layer was evaluated from the SEM micrographs with 500× magnification using Image-Pro Plus 5.0 software (Media Cybernetics Company, Silver Spring, MD, USA). For each specimen, at least three images were examined.

It must be noted that the alloy matrixes of all the four systems should have completely melted after heating for about 20 min at 993 K (720 °C) [9,10]. That is, they were not all in semisolid state. The reasons why this temperature was selected include two aspects. One is that all of the matrixes only contain pure Al when the alloying element contents are zero and there is no semisolid interval. The other is that, to guarantee the comparability of the experiment results between the different systems, it is best to maintain a given heating temperature for all of the specimens. In addition, the temperature of 993 K (720 °C) is usually employed as pouring temperature for Al alloys in both laboratory experiments and engineering productions. However, the aim of this work is to clarify the effects of the alloying elements on the reaction process, reaction products, and the microstructure of the resulting reinforcing particles during powder-thixoforming Al matrix composites with core-shell-structured Ti@compound_p_. So, experiments in semisolid states were also carried out to verify the results from the above liquid–state experiments. The green compacts of the Al–5Ti–5.5X (X = Si, Cu, Mg, and Zn) systems were heated for 30 min at semisolid temperatures of the corresponding matrixes, and then water–quenched. The employed heating temperatures for the Si–, Cu-, Mg- and Zn-containing compacts were 863 K (590 °C), 898 K (625 °C), 893 K (620 °C), and 923K (650 °C), respectively, at which the liquid phase fractions of all the four systems were about 0.5 according to the Al–Si, Al–Cu, Al–Mg, and Al–Zn binary phase diagrams [27]. The microstructure observation and phase-constituent analysis of the resulting reaction products were also carried out using the same techniques to the above liquid-heating states.

## 4. Results and Discussion

### 4.1. Effects of Alloying Elements on the Phase Constituents of Reaction Products

**Figure 4:**
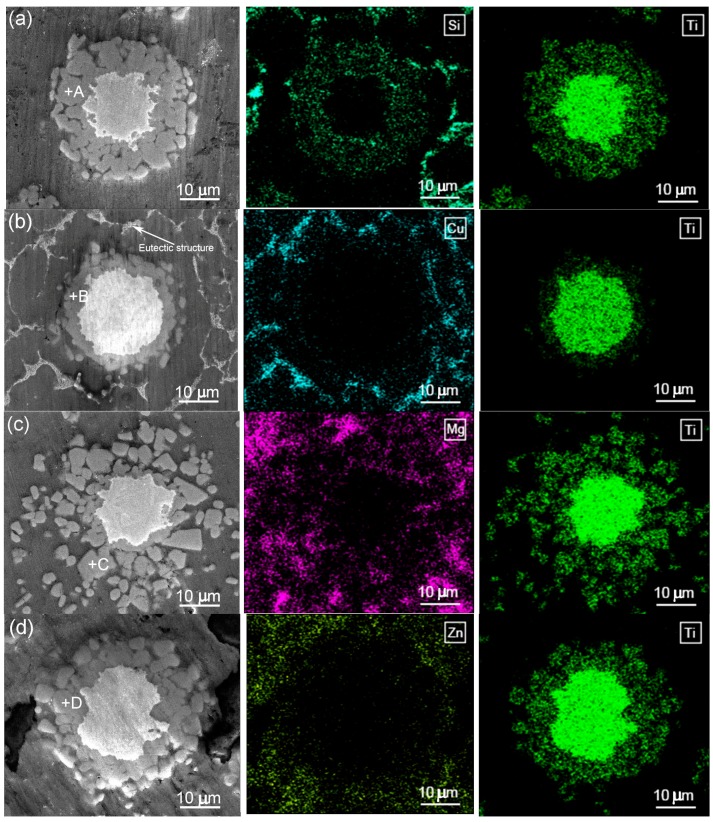
Scanning electron microscope (SEM) micrographs and energy dispersive spectroscope (EDS) maps of the reinforcing particles in the Al–5Ti–5.5X compacts heated at 993K (720 °C) for 30 min and then water-quenched. (**a**) X = Si; (**b**) X = Cu; (**c**) X = Mg; and, (**d**) X = Zn.

**Figure 5:**
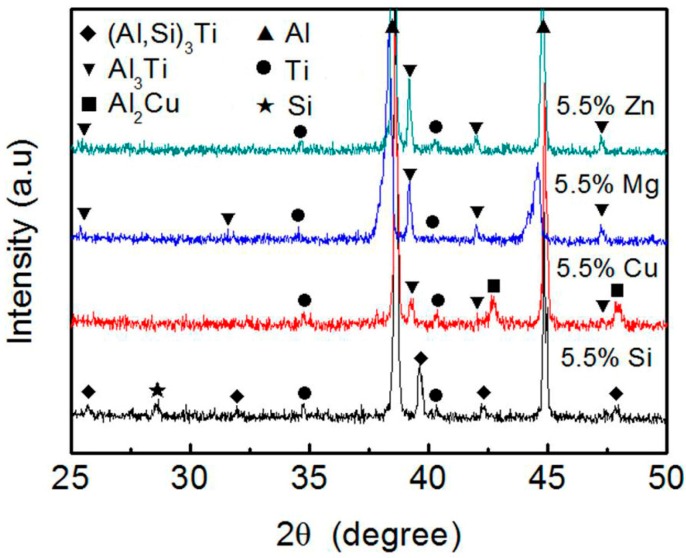
XRD patterns of the Al–5Ti–5.5X (X = Si, Cu, Mg, and Zn) compacts heated at 993K (720 °C) for 30 min and then water–quenched.

Figure 4 shows the SEM micrographs and EDS maps of the in situ formed reinforcing particles in the Al–5Ti–5.5X (X = Si, Cu, Mg and Zn) compacts heated at 993 K (720 °C) for 30 min. It reveals that Si uniformly distributed in the reaction layer besides in the eutectic structures of the Al matrix (Figure 4a), which demonstrates that Si actually participated in the reaction between Ti powders and Al melt and the reaction product contained Si element. The EDS quantitative analysis for point A marked in Figure 4a indicates that (Al + Si)/Ti atomic ratio is close to 3:1 (Table 5), being just consistent to the composition of (Al,Si)_3_Ti phase. This implies that the reaction product for the Si-containing system is the (Al,Si)_3_Ti phase. Cu element all basically segregated in the eutectic structures of the Al matrix, and there was no Cu element in the reaction product (Figure 4b). Mg and Zn distributed in both the primary Al grains and eutectic structures, and the reaction products also did not contain the corresponding alloying elements (Figure 4c,d). These results are consistent to the fact that the solubilities of Si and Cu in Al phase are smaller than those of Mg and Zn [27]. More importantly, the results indicate that Cu, Mg, and Zn did not actually participate in the reaction. In the all cases, Ti element was distributed in the reaction products besides in the residual unreacted Ti phase (the Ti maps in Figure 4). The EDS results for the reaction products (marked by B, C and D in Figure 4b–d respectively) in the Cu, Mg, and Zn-containing systems show that Al/Ti atomic ratios were all close to 3:1 (Table 5), which means that all of the reaction products in these three systems were Al_3_Ti phase, being exclusive of the other compounds. The results from XRD further confirm this conclusion, the Ti-having compound is Al_3_Ti phase for the Al–5Ti–5.5X (X = Cu, Mg, Zn) systems, while that is (Al,Si)_3_Ti phase for the Al–5Ti–5.5Si alloy (Figure 5). That is, Si element participated in the reaction between Ti powders and Al melt and a Al–Ti–Si ternary phase of (Al,Si)_3_Ti formed after the compact was heated for 30 min at 993 K (720 °C), while Cu, Mg, and Zn did not take part in the reaction and only one binary phase of Al_3_Ti generated.

Previous investigation proposed that Al atoms in Al_3_Ti phase could be partially substituted by Si atoms in Al–Si–Ti alloys, and Si content in the resulting (Al,Si)_3_Ti varied in a wide range and its maximum content was up to 15.07 at % [25]. In this work, the Si content is 9.9 at % and is within this range (Table 5). In addition, the radius of Si atom is smaller than that of Al atom [25], and thus the interplanar spacing and lattice constants of (Al,Si)_3_Ti phase should be smaller than those of Al_3_Ti phase and continuously decrease with the increase of Si content. According to Bragg diffraction formula [28], the diffraction peak of (Al,Si)_3_Ti phase will move towards the sites with a larger 2θ in XRD diffractogram. The XRD results demonstrate this deduction, the 2θ values corresponding to (Al,Si)_3_Ti phase in the Al–5Ti–5.5Si alloy are larger than those of the Al_3_Ti phase in the other systems (Figure 5).

It is just due to the replacement of Al in the Al_3_Ti phase by Si that there are several ternary compounds to possibly form in the Al–Ti–Si system, including AlTi_2_Si_3_ (τ2), AlTi_6_Si_3_, AlTi_4_Si_7_, Al_5_Ti_7_Si_12_ (τ1), and Al_12_Ti_5_Si_3_ [29]. The most acceptable chemical formula of τ1 phase can be expressed as (Ti_1–x_Al_x_)(AlySi_1–y_)_2_, where x ≤ 0.12 and 0.06 ≤ y ≤ 0.25 [30]. That is, the compositions of τ1 phase are estimated to be 8–20 at % Al, 29.3 at % Ti, and 62.6–50 at % Si. τ2 phase can be presented as Ti(Al_x_Si_1–x_)_2_, with the x of 0.15–0.3. As indicated above, the maximum solubility of Si in Al_3_Ti phase can be up to 15.07 at %. The crystal structure of this Si-containing Al_3_Ti phase is still maintained the tetragonal crystal structure of Al_3_Ti, so this phase is expressed as (Al,Si)_3_Ti [31]. But, it then transforms into τ1 phase when the Si content exceeds 15.07 at %. This is consistent with the above thermodynamic analysis, the stability of Al_3_Ti then decreases when the Si content exceeds 7 wt % and Al_3_Ti amount decreases as Si content in the Al–Ti–Si system increases. Another ternary phase, τ3, has been reported by Raman and Schubert, but its crystal structure and composition have not been identified [21]. So, it can be found that the ternary phases in the Al–Ti–Si system are significantly depended on the Si concentration.

**Figure 6:**
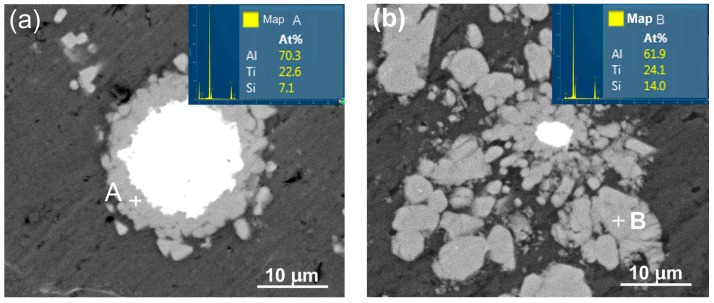
EDS analysis of reaction products in the (**a**) Al–5Ti–2Si and (**b**) Al–5Ti–9Si compacts heated for 30 min at 993 K (720 °C) and then water-quenched. The insets are the EDS maps for points A and B.

To further verify the effect of Si content on the ternary compounds, the experiment results from the Al–5Ti–2Si and Al–5Ti–9Si alloys heated for 30 min at 993 K are also presented. As shown by the inserts in Figure 6, the reaction products (marked by A and B in Figure 6a,b, respectively) in these two alloys were all (Al,Si)_3_Ti phase. A previous investigation indicated that the (Al,Si)_3_Ti compound was stable at 993K (720 °C) in Al–Ti–Si ternary system [31].This result at least states that all of the reaction products for the Al–Ti–Si system with Si contents ranging of 2–9 wt % only contain one compound of (Al,Si)_3_Ti at heating for 30 min.

**Figure 7:**
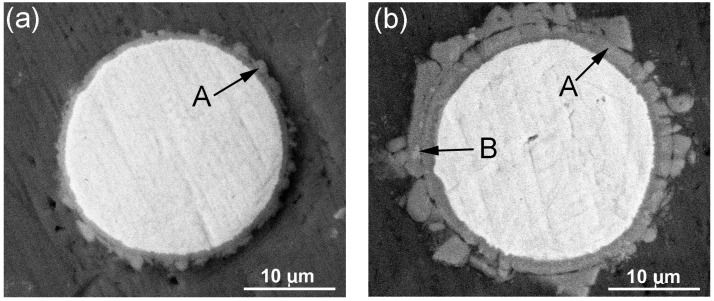

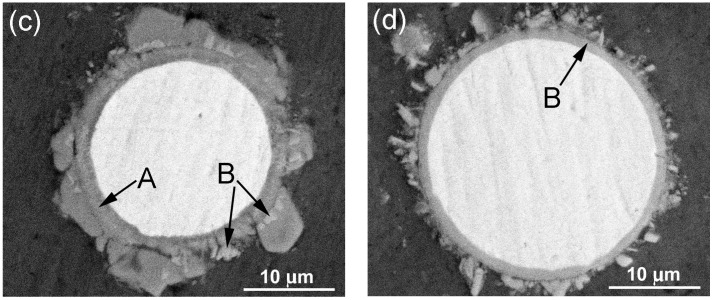
Micrographs of reinforcing particles in the Al–5Ti–XSi compacts heated at 993 K (720 °C) for 10 min and then water-quenched. (**a**) X = 2; (**b**) X = 5.5; (**c**) X = 7; and, (**d**) X = 9.

**Figure 8:**
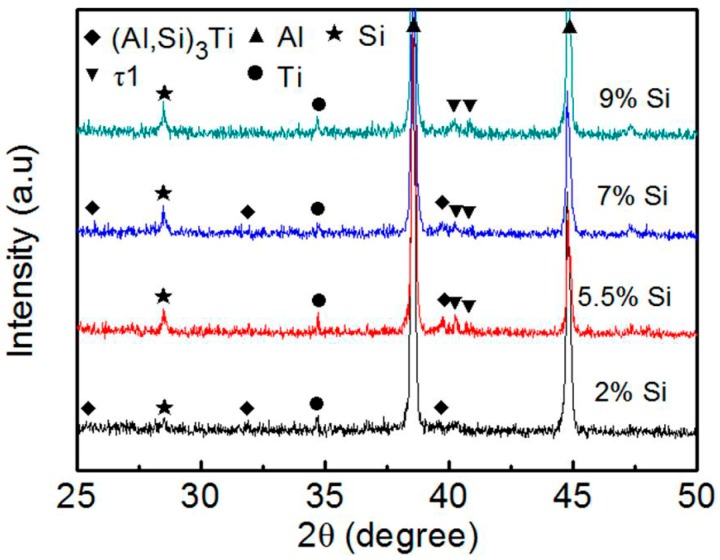
XRD patterns of the Al–5Ti–XSi (X = 2, 5.5, 7 and 9) compacts heated at 993 K (720 °C) for 10 min and then water-quenched.

Figure 7 shows the micrographs of the reinforcing particles in the Al–5Ti–XSi (X = 2, 5.5, 7, and 9) compacts heated for 10 min. It can be found that there were some white grey structures in the grey reaction products of the alloys with 5.5 and 9 wt % Si (marked by arrows B in Figure 7b,c), while the products in the other two systems only had unique-color phase (Figure 7a,d). In fact, the EDS results indicate that the products marked by arrows A in Figure 7 all belong to a Al–Ti–Si ternary phase with low Si concentration, while those marked by arrows B are a Al–Ti–Si ternary phase with high Si content (Table 6). When considering the errors of the EDS, it can be found that the (Al+Si)/Ti atomic ratios of all the A structures are close to 3:1, which implies that the A structures should be (Al,Si)_3_Ti phase, while the compositions of the B structures are basically within the ranges of τ1 phase compositions, 8–20 at % Al, 29.3 at % Ti, and 62.6–50 at % Si [30]. Namely, the product in the Al–5Ti–2Si alloy is the unique (Al,Si)_3_Ti phase, while that in the Al–5Ti–9Si alloy is the unique τ1 phase and those in the other alloys are a mixture of (Al,Si)_3_Ti and τ1 phases. This can be further demonstrated by the XRD result shown by Figure 8, there is only one Al–Ti–Si ternary phase of (Al,Si)_3_Ti for the alloy with 2 wt % Si, one ternary phase of τ1 for the alloy with 9 wt % Si and a two-phase mixture of (Al,Si)_3_Ti and τ1 for the other two alloys. In view of the present result, it can be deduced that the reaction product gradually transformed from (Al,Si)_3_Ti phase to τ1 phase as the Si addition increased from 2 wt % to 9 wt %. That is, high-content Si in the alloys can accelerate the formation of τ1 phase with high Si concentration.

For the Al–5Ti–5.5Si system, the reaction product is a mixture of (Al,Si)_3_Ti and τ1 phases when heated for 10 min, while it only contains one phase of (Al,Si)_3_Ti after being heated for 30 min. So, it can be deduced that the first formed product should be τ1 phase, and then the τ1 phase would gradually transformed into (Al,Si)_3_Ti as the heating proceeded. This deduction is consistent with the proposal that the (Al,Si)_3_Ti phase is thermodynamically stable at 993K (720 °C),while τ1 is a transitional or metastable phase [31]. As discussed above, the phase constituent of the reaction product is determined by Si content in it, and this Si content is highly depended on the Si concentration in the Al melt. The high former content should be supported by the high latter concentration. During heating, the first-formed liquid phase is resulted from the melting of residual Al–Si eutectics that have not dissolved into the primary Al phase in time [9]. For a hypoeutectic Al–Si alloy, such as the Al–Si matrix alloys that are used in this work, the first–formed eutectic liquid phase has high-content Si, which promotes the formation of reaction product with high Si content, i.e., τ1 phase. As the heating time increased, the Si content in the melt was decreased due to the consumption from the reaction and the partial melting of primary Al grains. Subsequently, part of Si atoms in τ1 phase dissolved into the liquid phase again in order to achieve a new equilibrium state, resulting in the transformation of τ1 into (Al,Si)_3_Ti. In addition, the newly-formed reaction product was also possibly (Al,Si)_3_Ti phase for the long–time heated compacts due to the obstacle of the compact and thick layer to Si diffusion towards the product/Ti interface and the decrease of Si concentration in the liquid phase. As shown by Figure 7b, c, the reaction products for the alloys heated for 10 min contain τ1 phase, but as illustrated by Figure 4a and Figure 6a,b, no τ1 phase can be found when heated for 30 min.

Summarily, Cu, Mg, and Zn elements did not participate in the reaction of Ti powders with Al melt and had no effect on the phase constituent of the reaction product. The reaction products of all the Al–Ti–X (X = Cu, Mg, and Zn) systems were the unique Al_3_Ti phase during the whole heating process. But, for the Al–Ti–Si system, Si element took part in the reaction. The first-formed product was a Al–Ti–Si ternary compound of τ1 phase with high Si concentration, and then the τ1 phase gradually transformed into another ternary compound of (Al,Si)_3_Ti phase with low Si content as the heating proceeded, or (Al,Si)_3_Ti phase directly formed due to the decrease of Si content in the Al melt and the obstacle of the thickened reaction shells to atom diffusion. The amount of τ1 phase and its existing time all increased as the Si content in the alloy increased because high-content Si could accelerate the formation of τ1 phase.

### 4.2. Effects of Alloying Elements on the Thickness of Reaction Shells

The aim of this work is to supply foundation for achieving core–shell structured Ti@compound_p_ with thick and compact compound shell during preparing Al matrix composites via powder thixoforming. So, the microstructure of the resulting reinforcements should be verified besides the phase constituents discussed above. The microstructure in this work mainly includes the thickness and compactness of the reaction shells.

**Figure 9:**
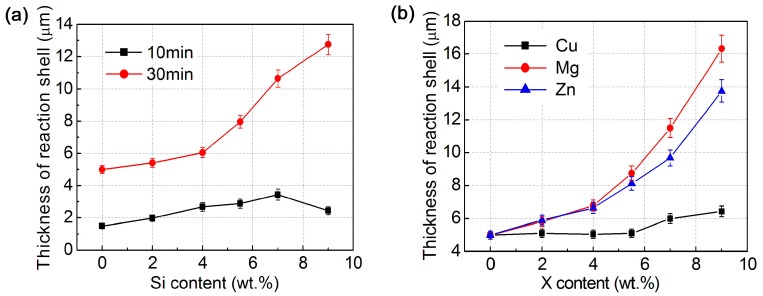
Variations of reaction shell thicknesses with (**a**) Si content in the Al–Ti–Si system heated at 993 K for different durations and (**b**) other alloying element X (X = Cu, Mg and Zn) contents in the Al–5Ti–5.5X systems heated at 993K(720 °C)for 30 min.

To accurately verify the effect of alloying elements on the thicknesses of the reaction shells, quantitative examinations were carried out, as shown by Figure 9. But, it can be found that the reaction products in most cases are not compact shells, but in agglomerates with different–sized irregular particles around the Ti cores (Figure 4, Figure 6 and Figure 7). The reason for leading to this result is attributed to the volume expansion from the reaction, which has been discussed in the following section. To examine the thickness, the amount of the reaction product was equivalent to a compact shell around a Ti core. Figure 9 indicates that the equivalent thicknesses all increased as the Si, Cu, Mg, and Zn contents increased for the corresponding systems heated for 30 min. That is, the increases in the alloying element contents all of the accelerated the reaction and enhanced the reaction rate. In contrast, the effect of Cu was very limited, and the thickness was almost invariable as its content increased from 2 wt % to 5.5 wt %, and only slightly increased when Cu content exceeded 5.5 wt % (Figure 9b). The effect of Mg was the largest and those of Si and Zn were considerable (Figure 9).

These phenomena can be visually verified by the presented micrographs. The shell thicknesses in the Al–5.5Ti–5X (X = Si, Mg, and Zn) systems were obviously larger than that in the Cu-containing alloy, those in the Si- and Zn-containing systems were seemly equivalent, but obviously smaller than that in the Mg-containing alloy (Figure 4). The effects of the alloying element contents can be seen by taking the micrographs of the Al–Ti–Si system as an example; only a thin shell formed for the 2 wt % Si alloy (Figure 6a), then significantly thickened as Si content increased (Figure 4a), and most of Ti has reacted and only a tiny core was left when Si content increased to 9 wt % (Figure 6b). But, for this system heated for 10 min, the thickness first increased as Si content increased from 2 wt % to 7 wt % and then decreased (Figure 9a). This change tendency can be clearly seen through comparing the micrographs shown in Figure 7. As discussed above, the reason for resulting in this difference from the case of heating for 30 min is contributed to the different microstructure compactness of the shells that originated from the different phase constituents. But, for the other systems, this phenomenon has not be found because the reaction phase did not change with the heating time and always maintained the unique phase of Al_3_Ti.

The results from the above thermodynamic calculations indicate that both the formation enthalpy and Gibbs free energy reduce as the Si content increases in a given range. This essentially means that Si element thermodynamically accelerates the formation of Al–Ti–Si ternary compounds in the Al–Ti–Si system. In view of dynamics, the reaction rate should be also promoted as the Si content increases because Si element is a participator of the reaction. So, the reaction shell thickened as the Si content increased for the cases of heating for 30 min (Figure 9a). The thermodynamic analysis also indicates that Mg, Cu, and Zn elements all can improve the stability of Al_3_Ti phase as their contents increase (Figure 2b), and thus accelerate the formation of Al_3_Ti phase and thickens the reaction shells. For a binary hypoeutectic Al alloy, the temperature at which eutectic liquid phase forms should lower as the solute (such as Mg, Zn, and Cu, also including Si) content increases during heating, and the amount of the resulting liquid phase must increase at a given temperature. It is expected that the contact of solid Ti particles with Al melt is closer, and the contact area is also larger than those with solid Al particles or less-amount Al melt. So, the reactions were accelerated and the shells thickened as the alloying element contents increased (Figure 9).

**Figure 10:**
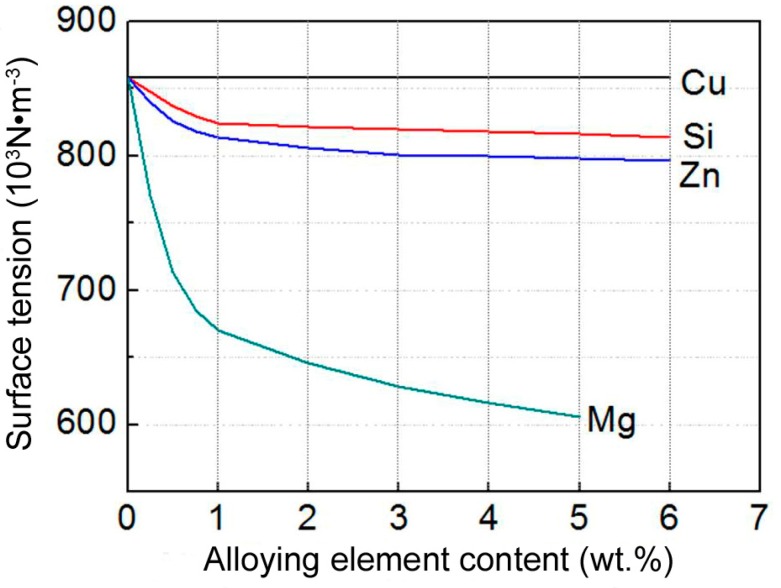
Relationships between the alloying element contents and surface tension of Al melt [33].

Furthermore, Mg is a surface-active element and can decrease the interfacial energy of solid–liquid interface [32]. Figure 10 presents the relationship between the alloying element contents and surface tensions of the corresponding binary aluminum alloy melts at temperatures of above liquidus 50–80K (50–80 °C) [33]. When compared with Si, Zn, and Cu, Mg element significantly decreases the surface tension of Al–Mg melt as its content increases. So, the wettability and thus the contact condition between the solid Ti particles and Al melt was improved, which accelerated the reaction and thickens the reaction shells.

The melting point of Zn is quite low (692.65K (419.65 °C) [34]), and the Zn powders will prematurely melt at the time of alloying with the Al powders, the Zn melt then accelerates the surrounding Al powders to dissolve into it to form Al melt. The more the Zn amount is, the more the generated Al melt amount is. So, the reaction was also promoted as the Zn content increased (Figure 9b).

Zhang et al. studied the microstructure and mechanical properties of an ultrafine-grained Al_3_Ti/Al–5.5Cu composite prepared by PM and pointed out that Al–Cu eutectic liquid phase formed when the composite compact was heated to 821 K (548 °C), and the liquid phase then penetrated into cracks in the Al_3_Ti layers to further participate in the reaction [35]. But Al–Cu binary phase diagram indicates that the maximum solubility of Cu in Al solid solution is up to 5.7wt % [34]. It is expected that most of Cu element might dissolve into the Al powders during the initial stage of heating due to the low content (5.5 wt %), and thus, eutectic liquid phase could not prematurely form. But, when the Cu content exceeded a given value (i.e., 7 wt %), liquid phase would form and its amount increased as the Cu content further increased. Based on this standpoint, the effect of Cu element shown in Figure 9b can be well interpreted. In addition, Figure 10 indicates that Si and Zn also slightly decrease the Al melt surface energy, and thus, the reactions are also promoted, but Cu element almost has no this effect.

That is, Si, Mg, Zn, and Cu all accelerated the reaction of Ti powders with Al matrix, and thus thickened the reaction shells as their contents increased. Comparatively, the effect of Mg was the largest, that of Cu was the smallest and only operated when its content exceeded 5.5 wt %, and Si and Zn had an equivalent middle effect. The common influence mechanism of the four elements is that they all promoted the premature formation of Al melt and the melt amount increased with increasing their contents. So, the contact condition between the Ti powders and Al matrix was improved because the contact was closer and the contact area was larger than those between the Ti powders and Al powders. However, the role of Si is also mainly ascribed to its participation in the reaction. Sometimes Si element decreased the shell thickness due to the variation of product phase constituents with Si content or heating time. The solubility of Cu in Al phase was quite high, and thus eutectic liquid phase formed till the Cu content reached a given value, so its effect did not carry out until the content exceeded 5.5 wt %. Mg could significantly decrease the interface energy between Ti powders and Al melt, and thus further improved the contact condition. But, for Zn element, increasing its content accelerated the premature formation of liquid phase due to its low melting point and the increase in the liquid amount.

### 4.3. Effect of Alloying Elements on the Microstructure Compactness of Reaction Shells

For the core-shell-structured Ti@compound_p_, the compound shells should be enough compact so as to play strengthening and toughening roles besides having a given thickness. But, the reaction products in most cases were in agglomerates with irregular and different-sized interconnected particles, or were in shells but contained small cracks (Figure 4, Figure 6 and Figure 7). As described above, the reason that resulted in these microstructures is the stress concentration from volume expansion occurred during transformation of Ti into compounds. It can be expected that the stress concentration is related to two main factors, one is the shell thickness and the other is the phase constituent.

The quantitative relationship between the stress concentration and the reaction shell thickness can be expressed as (taking forming Al_3_Ti shell as an example) [36]:

(3) $$\sigma_{Al_{3}Ti}=-\frac{E_{Al_{3}Ti}}{6\left(1-\upsilon_{Al_{3Ti}}\right)}\frac{t^{2}{}_{Al_{3}Ti}}{t_{Ti}}\left(\frac{1}{R}-\frac{1}{R_{0}}\right)$$

where $\sigma_{Al_{3}Ti}$ is the stress concentration in Al_3_Ti shell; $E_{Al_{3}Ti}$, $\upsilon_{Al_{3Ti}}$ are the modulus of elasticity and Poisson’s ratio of Al_3_Ti phase, respectively; $t_{Al_{3}Ti}$ is the thickness of Al_3_Ti shell and $t_{Ti}$ is the radius of residual Ti core; *R* and *R*_0_ are the radii of the reinforcing particle and the original Ti powder, respectively. It can be found that the thicker the shell thickness $t_{Al_{3}Ti}$ is, the larger the stress concentration $\sigma_{Al_{3}Ti}$ is, and thus the easier the shell fracture is. Our previous investigation on partial remelting of 2024Al–Ti green compact indicated that the stress could be up to 15.89 GPa when a Al_3_Ti shell with 2 μm thickness formed around a Ti powder with diameter of 9.28 μm, which is higher than the theoretical strength of Al_3_Ti phase (14.4 GPa) [10]. Under this condition, microcracks possibly generated within the shell. In addition, the stress is also concerned to the reaction rate for the shells with a same thickness; the stress concentration should increase as the reaction rate is quickened due to the decreased time for redistributing the stress. Of course, a higher reaction rate must result in a thicker shell in a given reaction time.

Based on the above discussion about the reaction shell thickness or reaction rate, it can be proposed that at a given heating time (i.e., 30 min) and a given element content (i.e., 5.5 wt %), the tendency to fracture of the reaction shell is the largest for the Al–Ti–Mg system, the least for the Al–Ti–Cu system, and equivalent middle for the Si- and Zn-containing systems. As shown by Figure 4, the reaction shell have fractured into the individual, different-sized particles for the Al–Ti–Mg system (Figure 4c), the shells for the Si- and Zn-containing systems are in the interconnected-particle agglomerates (Figure 4a,d), and that for the Al–Ti–Cu alloy is in a quite compact shell (Figure 4b).

In addition, for all of the systems, the shell thickness increased as the heating time was prolonged, and thus, the fracture tendency should be promoted with the heating time. This can be verified by Figure 7b and Figure 4a; for the Al–Ti–Si compact heated for 10 min, the reaction product is in a shell with small cracks (Figure 7b), but for the compact heated for 30 min, the product is in an interconnected particle agglomerate around the Ti core (Figure 4a). This can also be demonstrated by our previous investigations on the microstructure evolutions during partial remelting of A356–Ti and 2024Al–Ti compacts [9,10].

Similar to the effect of heating time, the increase in element content also enhances the fracture tendency due to the increased shell thickness. This can be seen through comparing Figure 7a,b; the reaction shell is quite compact for the 2 wt % Si-containing alloy, while cracks appeared as the Si content increased to 5 wt %. But, for the cases with 7 and 9 wt % Si, they did not obey this tendency due to the variation of phase constituent, which will be discussed as following.

As indicated above, the stress concentrations in different compounds are also different for the Al–Ti–Si system. This is resulted from the different volume expansion ratios for forming different compounds. The volume expansion ratio, Δ*V*, occurred during reaction can be expressed by the equation [36]:

(4) $$\Delta V=\frac{V_{Products}-V_{Reactants}}{V_{Reactants}}$$

where $V_{Products}$ and $V_{Reactants}$ are the total atomic volumes of reaction products and reactants, respectively. For this work, the products include Al_3_Ti, (Al,Si)_3_Ti, and τ1 phases, while the reactants only refer to pure Ti. According to the densities of Ti, Al_3_Ti, and τ1 phases [9,10,21], their molar volumes can be achieved as shown by Table 7. The volume expansion ratios of Ti transforming into Al_3_Ti and τ1 phases, $\Delta V_{Ti-Al_{3}Ti}$ and $\Delta V_{Ti-\tau 1}$, can be calculated by using Equation (4). The result shows that the $\Delta V_{Ti-Al_{3}Ti}$ and $\Delta V_{Ti-\tau 1}$ are 2.55 and 1.8, respectively (Table 7). That is, the volume is expanded by 2.55 and 1.8 times when Ti transforms into Al_3_Ti and τ1 phases respectively, and the volume expansion for forming Al_3_Ti phase is significantly larger than that for forming τ1 phase. But for the (Al,Si)_3_Ti phase, its density cannot be determined because of the uncertainty of its composition (the Si content varies in a wide range up to 15.07 at % [25]), and thus, the corresponding volume expansion ratio cannot be calculated. However, it is certain that its volume expansion ratio, $\Delta V_{Ti-{\left(Al,Si\right)}_{3}Ti}$, must be smaller than the $\Delta V_{Ti-Al_{3}Ti}$, but larger than the $\Delta V_{Ti-\tau 1}$ according to the Si contents in these phases. That is, the stress concentration in (Al,Si)_3_Ti phase is between those in τ1 and Al_3_Ti phases.

The reaction shells in the Al–Ti–Si system usually have two phases of (Al,Si)_3_Ti and τ1, and the proportion of τ1 phase decreased as the heating time prolonged and increased with increasing Si content, while those of the other three systems always have a single phase of Al_3_Ti. So, it is suggested that the stress concentrations, and thus the shell compactness for the Cu-, Mg-, and Zn-containing systems are only determined by the shell thickness or reaction rate, as discussed above. But, for the Al–Ti–Si system, the stress state and the resulting shell compactness are also affected by the phase constituent besides the shell thickness.

At a given shell thickness, the reaction shells in the Al–Ti–Si system should be more compact than those in the other three systems due to the lower stress concentration in (Al,Si)_3_Ti and τ1 phases, and will become more and more compact with shortening heating time or increasing Si content because of the increased τ1 phase. As shown by Figure 7b, the reaction product in the 5.5 wt % Si-containing system heated for 10 min is a relatively compact shell, but as shown by Figure 4a, the shell have fractured into an agglomerate when the heating time increased to 30 min. It should be noted that the fracture is also partially contributed to the increased shell thickness. Increasing Si content not only thickens the shells, but also increases the proportion of τ1 phase. The former promotes the fracture of the shells, which can be verified by comparing Figure 7a,b, as described above, also can be seen through comparing Figure 4a and Figure 6a,b, the product in the 2 wt % Si system is in a quite compact shell, that in the 5.5 wt % Si alloy have fractured into the interconnected particles and the fracture degree became more serious when the Si content increased to 9 wt %. But, the latter improves the compactness, which can be confirmed by comparing Figure 6b–d, the shell for the 9 wt % Si system is quite compact, while those of the 5.5 wt % and 7 wt % Si systems contain cracks. It is just due to the obstacle of the compact shell to the reaction that the shell in the 9 wt % Si system is quite thin (Figure 7d). That is, decreasing the heating time and increasing Si content are beneficial to obtain compact reaction shells, but the former parameter and sometimes the later parameter all decrease the shell thickness. Therefore, to achieve the desirable core-shell-structured Ti@compound_p_ with a compact and thick compound shell, increasing Si content is generally helpful, but a suitable heating time must be coupled.

Generally, the microstructure compactness of the reaction shells for the Al–Ti–X (X = Cu, Mg, and Zn) systems is only depended on the shell thickness, and become more and more loose as the Mg, Cu, and Zn contents or heating time increase due to the enhanced stress concentrations from the increased shell thickness. The influence degrees of these three elements are same to those on the shell thickness. But, for the Al–Ti–Si system, the compactness is also related to the phase constituent besides the shell thickness because of its dual-phase characteristic of the resulting reaction shells. The stress concentration generated in (Al,Si)_3_Ti phase is larger than that inτ1 phase, but smaller than that in Al_3_Ti phase. So, the shells of the Al–Ti–Si system usually are more compact than those of the other systems when the shell thicknesses are same. Increasing Si content is beneficial to obtain ideal core-shell-structured Ti@compound_p_ with a thick and compact compound shell, but simultaneously, a suitable heating time must be coupled.

### 4.4. Microstructures of the Reaction Shells in Semisolid Compacts

**Figure 11:**
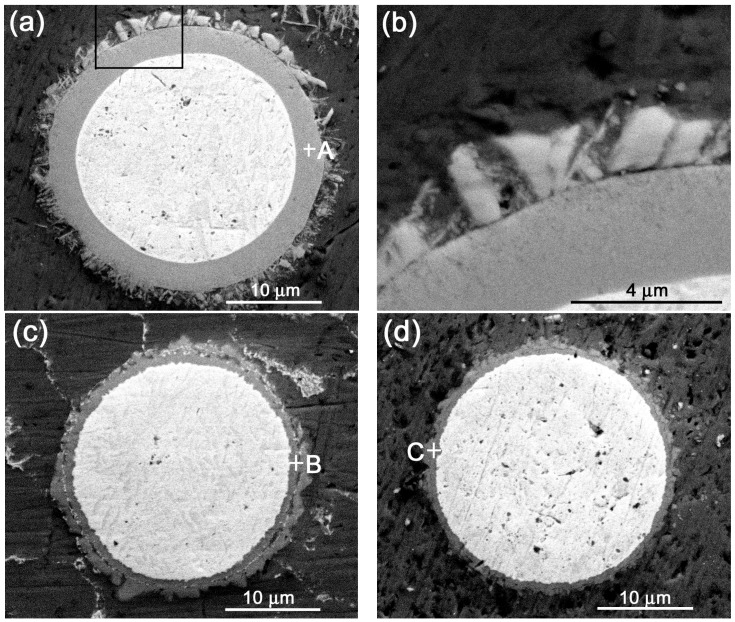
Micrographs of reinforcing particles in the Al–5Ti–5.5X compacts heated for 30 min at corresponding semisolid temperatures and then water-quenched. (**a**) X = Si, 863 K (590 °C) and (**b**) the enlarged view of the zone marked by rectangle in (**a**); (**c**) X = Cu, 898 K (625 °C); and, (**d**) X = Zn, 923 K (650 °C).

**Figure 12:**
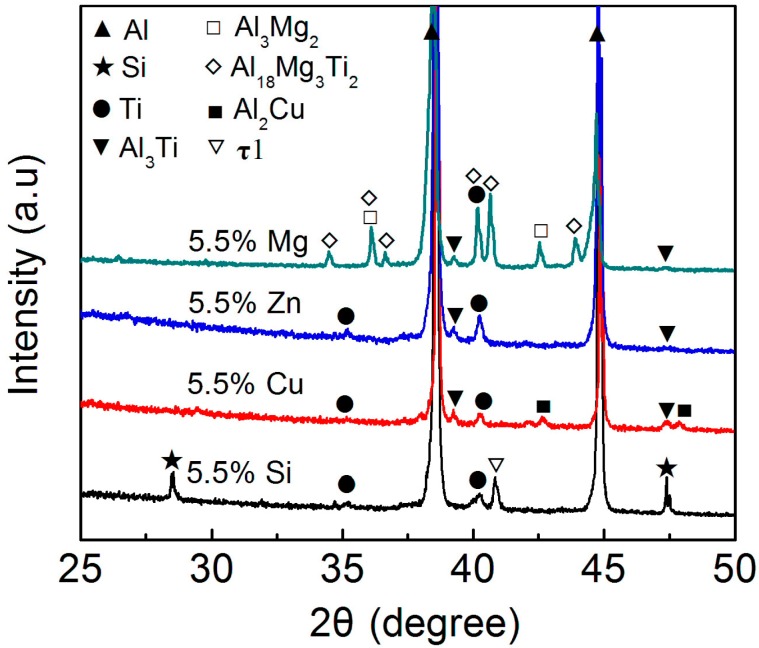
XRD patterns of Al–5Ti–5.5X (X = Si, Cu, Zn, and Mg) compacts heated at corresponding semisolid temperatures for 30 min and then water-quenched.

Figure 11 shows the microstructures of reinforcements in the semisolid Al–5Ti–5.5X (X = Si, Cu, and Zn) compacts. It can be seen that the reaction product in the Si–containing system is a homogeneous shell with a thickness of about 3.7 μm except the outside jagged structures (Figure 11a) and the shell is quite compact (Figure 11b). In addition, unlike with those shown by Figure 7b,c, the shell only contains one uniform–gray compound. The EDS result indicates that the composition of this compound is consistent to that of τ1 (Al_5_Ti_7_Si_12_) phase (location A in Table 8) [30], and the XRD results shows that there is only one Ti-containing compound of τ1 phase (Figure 12). These mean that the reaction product in this case is unique τ1 phase and the transformation of τ1 phase into (Al,Si)_3_Ti phase has not operated. It is known that the volume expansion for forming τ1 phase is the smallest, so the resulting product is in a compact shell (Figure 11a). That is, ideal core-shell-structured Ti@τ1 reinforcing particles were obtained after partial remelting of the Al–Ti–Si compact.

Comparatively, the shell in the Cu-containing system is quite thin (about 1.5 μm), although it seems also quite compact (Figure 11c). Figure 11d shows that the status of the Zn-having system is very similar to that of the Cu-containing alloy, but the shell thickness is rather thinner (about 1μm). The products in these two systems are all in a gray-color structure. The Al/Ti atomic ratios are basically identical and approximately equal to 3:1 (locations B and C in Table 8). In addition, the XRD result also indicates that these two systems only have one Ti-having compound of Al_3_Ti (Figure 12).These imply that the reaction products in the Cu-and Zn-containing systems are all Al_3_Ti phase, similar to those in the liquid cases discussed above.

**Figure 13:**
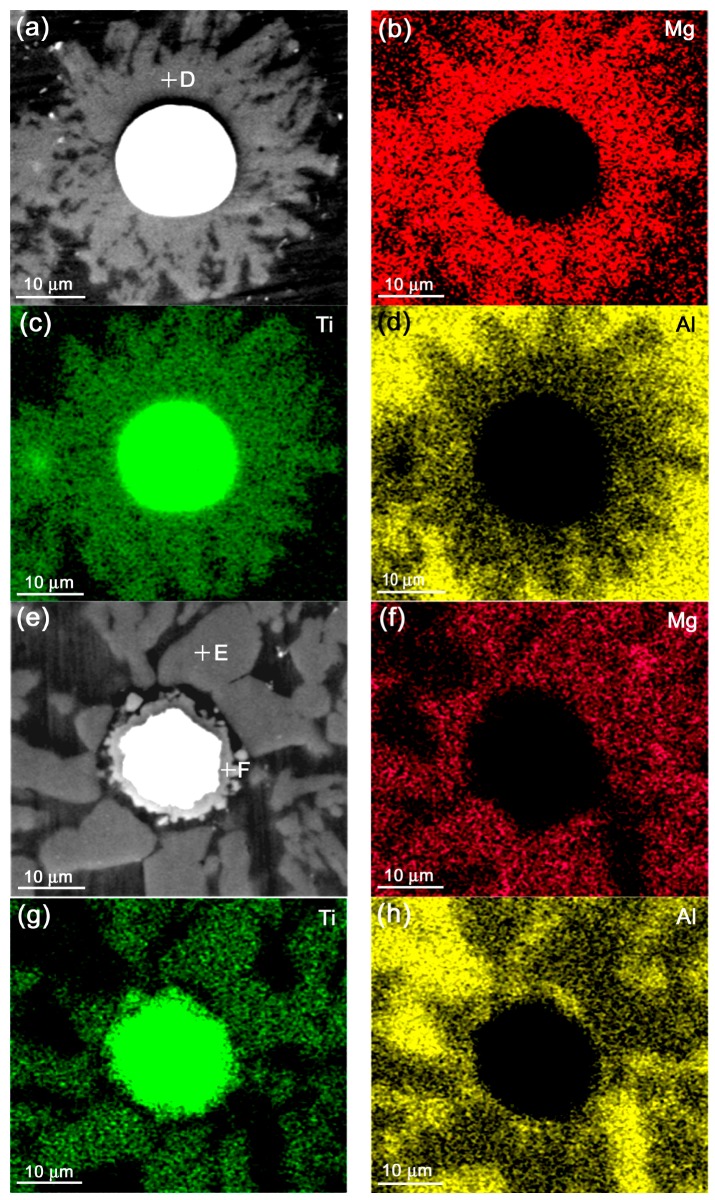
Micrographs and EDS maps of reinforcing particles in the Al–5Ti–5.5Mg compact heated for 30 min at 893 K (620 °C),and then water-quenched. (**a**,**e**) micrographs, (**b**,**f**) Mg maps, (**c**,**g**) Ti maps, (**d**) and (**h**) Al maps.

But, for the Mg-containing alloy, the result is different from that of the liquid-heating state. There are two kinds of situations in the semisolid state in view of the resulting reinforcement morphologies. One is that the reinforcement is composed of a Ti core and surrounding compound strips that radially distribute around the Ti core (Figure 13a). More importantly, the compound is rich in Mg element (Figure 13b) besides containing Ti (Figure 13c) and Al (Figure 13d) elements. The other is as shown by Figure 13e, the reinforcement is consisted of a Ti core, a compact compound shell and the surrounding polygonal particles that are obviously separated by a matrix alloy layer from the center core-shell-structured particle. The EDS results indicate that the polygonal particles, similar to the compound strips in Figure 13a, are rich in Mg and Ti elements besides containing Al, while the compact shell is rich in Ti and Al, and does not include Mg element (comparing Figure 13e–h). The quantitative examinations from EDS show that both the strip- (point D in Figure 13a) and particle-like (point E in Figure 13e) compounds have the same composition (locations D and E in Table 8), which is coincident with the composition of Al_18_Mg_3_Ti_2_ternary compound [37,38,39], while the shell-like compound (point F in Figure 13e) has a Al/Ti atomic ratio of 3:1 (location F in Table 8), which is just consistent to that of Al_3_Ti phase. The XRD results also present that there are two kinds of Ti-containing compounds in this system, Al_18_Mg_3_Ti_2_ and Al_3_Ti (Figure 12). That is, the out polygonal particles are Al_18_Mg_3_Ti_2_ phase and the inner shell is Al_3_Ti phase. In view of the relative distribution sites of these two compounds with the Ti core, it can be proposed that the first formed reaction product with Ti was Al_3_Ti phase, and then the Al_3_Ti phase gradually transformed into Al_18_Mg_3_Ti_2_ phase during the subsequent heating. Namely, the Al_3_Ti compound that was first formed during heating at the semisolid temperature is a transitional phase in the Al–Ti–Mg system and the final stable product is the ternary compound of Al_18_Mg_3_Ti_2_. According to this standpoint, it is expected that there should also be an Al_3_Ti ring around the Ti core in Figure 13a, but it may be too thin to see it at the present conditions.

Similar to that at 993 K (720 °C), the preferential formation of Al_3_Ti at the semisolid temperature of 893 K (620 °C) is also attributed to the large affinity between Ti and Al atoms for the Al–Ti–Mg system. But, the subsequent transformation of the first formed Al_3_Ti into Al_18_Mg_3_Ti_2_ phase should be resulted from the smaller thermostability of Al_3_Ti than that of Al_18_Mg_3_Ti_2_. The existing investigations indicated that the reaction product between solid Ti particles and Al–Mg matrix alloys was the unique Al_3_Ti phase at above liquidus temperatures of the Al–Mg matrix alloys, but an Al_18_Mg_3_Ti_2_ phase ring gradually formed around the Al_3_Ti particles during annealing treatments at 723–820 K (450–547 °C), and all of the Al_3_Ti particles would completely transform into the Al_18_Mg_3_Ti_2_ particles after being enough annealed [37,38,39]. In addition, during laser-welding of AZ31B Mg alloy and 6061 Al alloy using pure Ti interlayer, a small amount of Al_18_Mg_3_Ti_2_ phase generated at the edge regions of the interfacial layer close to AZ31B alloy and the other regions were occupied by Al_3_Ti phase [40].This implies that Al_18_Mg_3_Ti_2_ phase only formed in the edge regions in which the temperatures were relatively low, while Al_3_Ti phase generated in the central regions with high temperatures during welding. This also indicates that the thermostability of Al_3_Ti phase is lower than that of Al_18_Mg_3_Ti_2_ phase at low temperatures for the Al–Ti–Mg system.

Figure 13a,d shows that only a small-sized Ti core was left and a larger amount of compounds have generated in the Mg-containing system, regardless of the product morphology. When compared with the other three systems (Figure 11a–d), it can be found that the acceleration roles to the reaction decrease in a sequence of Mg, Si, Cu, and Zn. That is, Mg also has the largest acceleration role, but the effect of Zn was sharply decreased as compared with that of the liquid-heating state. The acceleration effect of Mg can also be found in the other investigations, both the Ti content and reaction temperature needed for forming Al_3_Ti are decreased in Mg-containing Al alloys [32,39]. In fact, the acceleration effects of all the four elements were decreased to different degrees at the semisolid states due to the decreased temperatures (comparing the corresponding metallographs in Figure 11and Figure 13 with those in Figure 4).

According to the Al–Zn binary phase diagram, the solubility of Zn in Al solid solution is very large (higher than 80 wt %) [27]. So it can be expected that Zn atoms in the prematurely formed Zn liquid during the initial stage of partial remelting would subsequently dissolve into the surrounding Al powders, which led the liquid phase amount to decrease, and thus the acceleration affect on the reaction was reduced in a certain time period. But, for the case of liquid-heating state, most of Zn atoms in the first-formed Zn melt did not dissolve into the Al powders because the Al powders should also quickly begin melting due to the high heating temperature (993 K (720 °C)). That is, the effect of the dissolution of Zn atoms on the liquid amount is quite small or the time for maintaining small-amount liquid is very short, and thus its effect on the reaction is also small.

In fact, it is just due to the same reason that the effect of Cu was also obviously decreased at the semisolid temperature of 898 K (625 °C) (comparing Figure 4b and Figure 11c), only the decrease range is smaller than that of Zn element. So, it is proposed that the effect of an alloying element on the formation of liquid phase, and thus on the reaction rate is related to its solubility in Al phase and heating temperature. Finally, in view of the morphologies of the reinforcements, it can be found that the desirable core-shell-structured Ti@compound reinforcing particles with compact and thick compound shell only can be achieved during partial remelting of the Al–Ti–Si compact.

In summary, most of the results from the liquid–state experiments are consistent to those from the semisolid state ones except the product phase constituent in the Al–Ti–Mg system and the reaction rate in the Al–Ti–Zn system. The first reaction product with Ti in Al–Ti–Mg system was still Al_3_Ti phase at the semisolid state, but it then transformed into an Mg–Ti–Al ternary compound of Al_18_Mg_3_Ti_2_ possibly due to its smaller thermostability at this temperature. The reaction rates of all the four systems were obviously decreased at the semisolid states due to the decreased temperatures, but the decrease range in the Al–Ti–Zn system was so large that the resulting reaction rate became smaller than that in the Al–Ti–Cu system. The reason for leading to this result should be contributed to the large solubility of Zn in Al phase. More importantly, the desirable core-shell-structured Ti@compound_p_ can only be obtained during partial remelting of the Al–Ti–Si compact.

## 5. Conclusions

1. Thermodynamic analysis form Miedema’s model and JMatPro indicates that Si participates in the reaction between Ti powders and Al melt and promotes the formation of Al–Ti–Si ternary compounds, while Cu, Mg, and Zn do not involve the reaction and facilitate the formation of Al_3_Ti phase to different degrees.
2. The results from experiments about heating powder-mixture compacts at 993 K (720 °C) demonstrate the above thermodynamic proposals. For the Al–Ti–Si system, The first-formed product was a Al–Ti–Si ternary phase of τ1 with high Si content, and then it gradually transformed into (Al,Si)_3_Ti phase with low Si content. The proportion and existing time of τ1 phase all increased as the Si content increased. But, for the Al–Ti–X (X = Cu, Mg, and Zn) systems, the reaction products always contained one phase of Al_3_Ti.
3. Increasing Si, Mg, Zn, and Cu contents all thickened the reaction shells. In contrast, the effect of Mg is the largest, that of Cu is the smallest, and Si and Zn have an equivalent middle effect. But sometimes Si element decreased the thickness because of the variation of phase constituent with Si content or heating time. The reasons for leading to these results include their effects on the liquid phase formation, surface energy of Al melt, whether participating in the reaction and phase constituents.
4. The compactness of the reaction shells for the Al–Ti–X (X = Cu, Mg, and Zn) systems was only depended on the shell thickness, and usually became more and more loose as the element contents or heating time increased due to the enhanced stress concentration. But, for the Al–Ti–Si system, the compactness was also related to the phase constituent. The stress concentration generated in (Al,Si)_3_Ti phase was smaller than that in Al_3_Ti phase, but larger than that in τ1 phase. So, the shells in the Al–Ti–Si system were more compact than those in the other systems and Si element was beneficial to obtain ideal core-shell-structured Ti@compound_p_ with thick and compact compound shell.
5. Most of the above results from the liquid-heating state experiments were consistent to the semisolid state ones except the product phase constituent in the Al–Ti–Mg system and the reaction rate in the Al–Ti–Zn system. The first reaction product in the Al–Ti–Mg system was still Al_3_Ti phase, but it then transformed into an Mg–Ti–Al ternary compound of Al_18_Mg_3_Ti_2_. The decrease range of reaction rate in the Al–Ti–Zn system at semisolid state was so large that the resulting reaction rate became smaller than that in the Al–Ti–Cu system. The desirable core-shell structured Ti@compound_p_ were only obtained in the semisolid Al–Ti–Si system.

## Figures and Tables

**Table 1:** The parameter values used in Miedema’s model [18].

Element	$n_{ws}^{1/3}\left({\left(du\right)}^{1/3}\right)$	φ(V)	$V^{2/3}\left({\mathrm{cm}}^{2}\right)$	μ	r/p
Al	1.39	4.20	4.6	0.07	1.9
Si	1.50	4.70	4.2	0.04	2.1
Cu	1.47	4.55	3.7	0.07	0.3
Mg	1.17	3.45	5.8	0.10	0.4
Zn	1.32	4.10	4.4	0.04	1.4
Ti	1.47	3.65	4.8	0.04	1.0

**Table 2:** Gibbs free energies for forming Al–Ti compounds [21].

Compound	Function with Temperature T	Gibbs Free Energy at 993K (720 °C) (J/mol)
AlTi_3_	−29633.6 + 6.70801T	−22972.546
AlTi	−37445.1 + 16.79376T	−20718.515
Al_2_Ti	−43858.4 + 11.02077T	−32914.775
Al_3_Ti	−40349.6 + 10.36525T	−30056.907
Al_5_Ti_2_	−40495.4 + 9.52964T	−31032.467

**Table 3:** The characteristics of the raw powers used in this work.

Powder	Purity	Average Grain Size/μm	Preparation Method
Al	99.9%	44	Atomization
Ti	99.8%	17.19	Atomization
Si	99.7%	550	Mechanical crushing
Cu	99.5%	23	Electrolytic method
Mg	99.7%	325	Atomization
Zn	99.5%	13	Atomization

**Table 4:** The constituents of powder mixtures used in this work.

	Content (wt %)					
	Ti	Si	Cu	Mg	Zn	Al
Effect of Si element	5	2, 4, 5.5, 7, 9	0	0	0	Balance
Effect of Cu element	5	0	2, 4, 5.5, 7, 9	0	0	Balance
Effect of Mg element	5	0	0	2, 4, 5.5, 7, 9	0	Balance
Effect of Zn element	5	0	0		2, 4, 5.5, 7, 9	Balance

**Table 5:** EDS results of the reaction products marked by A, B, C, and D in Figure 4.

Location	Composition (at %)					
	Al	Ti	Si	Cu	Mg	Zn
A	67.6	22.5	9.9	–	–	–
B	75	25	–	0	–	–
C	75.5	24.5	–	–	0	–
D	75.6	24.4	–	–	–	0

**Table 6:** EDS results of the reaction products marked by A and B in Figure 7.

Location	Composition (at %)		
	Al	Ti	Si
A in Figure 6a	63.8	23.4	12.8
A in Figure 6b	54.2	24.3	21.5
B in Figure 6b	21.2	29.6	49.2
A in Figure 6c	52.2	25.3	22.5
B in Figure 6c	20.8	28.9	50.3
B in Figure 6d	22.9	27.3	49.8

**Table 7:** Densities and molar volumes of Ti, Al_3_Ti, and τ1 phases and corresponding volume expansion ratios.

Phases	Density(g/cm^3^)	Molar Volume (cm^3^/mol)	Volume Expansion Ratio
Ti	4.504	10.628	–
Al_3_Ti	3.414	37.730	2.55
τ1 (Al_5_Ti_7_Si_12_)	3.874	208.313	1.8

**Table 8:** EDS results of the reaction products marked by A–F in Figure 11 and Figure 13.

Location	Composition (at %)					
	Al	Ti	Si	Cu	Mg	Zn
A	17.4	31.7	50.9	–	–	–
B	74.4	25.6	–	0	–	–
C	75.2	24.8	–	–	–	0
D	80.4	8.8	–	–	10.8	–
E	80.5	9.9	–	–	9.6	–
F	74.2	25.8	–	–	0	–
